# AI-Based Automation for Medication Reconciliation: Scoping Review

**DOI:** 10.2196/86760

**Published:** 2026-05-11

**Authors:** Juan Pablo Tabja Bortesi, Maria P Becerra, Jonathan Ranisau, Bonnie Wen, Praveen Nadesan, P J Devereaux, Michael McGillion, Jeremy Petch

**Affiliations:** 1 Centre for Data Science and Digital Health, Hamilton Health Sciences, Hamilton, ON, Canada; 2 School of Epidemiology and Public Health, University of Ottawa, Ottawa, ON, Canada; 3 Faculty of Health Sciences, McMaster University, Hamilton, ON, Canada; 4 Michael G. DeGroote School of Medicine, Faculty of Health Sciences, McMaster University, Hamilton, ON, Canada; 5 Population Health Research Institute, Hamilton, ON, Canada; 6 Division of Cardiology, Department of Medicine, McMaster University, Hamilton, ON, Canada; 7 School of Nursing, Faculty of Health Sciences, McMaster University, Hamilton, ON, Canada; 8 Institute for Health Policy, Management and Evaluation, University of Toronto, 155 College St 4th Floor, Toronto, ON, M5T 3M6, Canada, 1 4164769039

**Keywords:** medication reconciliation, artificial intelligence, AI, health IT, machine learning, patient safety, continuity of care

## Abstract

**Background:**

Medication reconciliation (MedRec) has the potential to improve patient safety by enhancing the continuity of medication information across settings. MedRec involves 3 core tasks: the creation of a best possible medication history, the identification of medication discrepancies among medication lists, and the resolution of medication discrepancies. While artificial intelligence (AI) has the potential to improve MedRec, existing reviews have not identified the ways in which researchers have used AI to facilitate MedRec tasks and their constituent subtasks or the level of automation achieved.

**Objective:**

This scoping review aimed to map how previous research has applied AI to MedRec tasks and subtasks and assess the extent of automation achieved.

**Methods:**

We searched MEDLINE, Embase, Web of Science, IEEE Xplore, and Compendex in June 2024 for studies that used AI to support a MedRec task or subtask, excluding entirely rule-based tools or studies focused on other aspects of medication management. After screening 2345 unique records, we conducted backward citation searching of studies included at the full-text stage, identifying an additional 795 unique records. We used a 4-stage model of human information processing as a structural lens to guide our considerations of automation, mapping the core tasks of MedRec onto this model.

**Results:**

A total of 94 studies met the inclusion criteria. All studies addressed subtasks related to the creation of a best possible medication history. Only 2.1% (n=2) of the studies also addressed the identification of discrepancies. Thus, the highest stage of automated information processing achieved was information analysis, although most studies (92/94, 97.9%) only automated information acquisition steps. Most studies (67/94, 71.3%) used free-text clinical notes from the electronic health record, although a significant proportion (21/94, 22.3%) used images of pills or images of other medication-related items. Studies using text-based data used a variety of machine learning methods (eg, recurrent neural networks, conditional random fields, support vector machines, and transformers), whereas those that leveraged images typically used convolutional neural networks. Most studies (61/94, 64.9%) used publicly available data from benchmarking datasets (eg, n2c2 2022) and were strictly model development studies, with only 1.1% (n=1) being usability studies.

**Conclusions:**

This is the first review to consider the role of AI in the automation of MedRec tasks, offering a basis for prioritizing future development efforts. Current applications of AI to automate MedRec tasks are preliminary, with most work focusing on the extraction of medication information and limited to proof-of-concept model development. Future work should consider addressing infrastructural barriers to the AI-based automation of MedRec tasks (eg, data incompleteness in sources of medication information) and exploring approaches to automate discrepancy resolution. Beyond developing models, there is also a need to implement them in tools and evaluate them in real-world contexts.

## Introduction

There is a potential for error at every stage of the medication use cycle, which can often lead to serious harms for patients. This avoidable harm is substantial: across care settings, a substantial proportion of adverse drug events (ADEs) have been estimated to be preventable, although estimates vary by context and definition [1-3]. The economic impact of ADEs is considerable, with estimates exceeding hundreds of millions of dollars per year across acute and primary health care systems [4]. Given the scale of the problem, in 2017, the World Health Organization announced *Medication Without Harm* as its third Global Patient Safety Challenge, outlining strategies to reduce avoidable medication-related harm [5]. One of the chief causes of medication errors is inaccuracies in medication lists used throughout a patient’s care.

Medication reconciliation (MedRec) is an important procedure for identifying such inaccuracies. MedRec generally includes the creation of a best possible medication history (BPMH) that is compared to a list of medications ordered by a health care professional (eg, a physician, nurse, or pharmacist) to address unintended discrepancies [6]. This typically occurs during care transitions, such as hospital admission and discharge, when medication errors frequently occur [7-10]. MedRec can also be performed in primary care or outpatient settings, often with patients at risk of readmission following discharge [11]. Recognizing the complexity of care transitions, organizations such as Accreditation Canada and the National Institute for Health and Care Excellence recommend enhanced MedRec to improve medication safety [12-15].

Previous research suggests that MedRec can be effective in identifying medication discrepancies, although evidence of improvements in clinical or health care use outcomes is mixed. A systematic review on the effectiveness of hospital-based, pharmacist-led MedRec found that MedRec was associated with significant reductions in all-cause hospital readmissions and all-cause emergency department visits despite heterogeneity across studies [16]. In contrast, a similar review found no significant reduction in health care use [17]. A Cochrane review evaluating the effect of MedRec on medication discrepancies, patient‐related outcomes, and health care use concluded that the certainty of the evidence in favor of MedRec was very low, noting heterogeneity across studies in intervention implementation, outcome definitions, and intervention effects [18]. The complexity and need for multiple coordinated components in MedRec interventions may explain their inconsistent outcomes [19,20]. These workflows are often time- and labor-intensive [10,21], which can compromise fidelity.

There have been efforts to develop electronic MedRec tools over the last 2 decades. These tools have various functionalities that may reduce the time and labor demands of traditional, paper-based MedRec, such as interfaces that visually facilitate comparison of medication lists [22] and automated retrieval of community medication lists [23]. Other efforts have focused on methods for conducting MedRec tasks remotely, such as creating an interactive web form or leveraging video visits [24-26]. Due to the need for repetitive data entry, MedRec workflows are ideal targets for artificial intelligence (AI)–driven automation [27]. Although automation has not been the focus of previous literature reviews of MedRec tools, Marien et al [28] conducted a systematic review of electronic MedRec tools and found that only half of the identified tools provided automated discrepancy detection. Ciudad-Gutiérrez et al [29] reviewed a similar set of electronic MedRec tools, noting that approximately half could automatically retrieve medication information. While both functionalities involve important processes that could be automated, no review has yet considered the role of AI in automating MedRec. AI has recently been applied to specific subtasks within the broader workflows of MedRec (eg, extracting medications from free-text notes [30]), but previous literature reviews of MedRec tools have not explored how AI has been leveraged to facilitate MedRec tasks and subtasks. Existing reviews have also examined the use of AI for other aspects of medication use and safety, such as ADE prediction and detection [31], or more broadly for medication management [32,33], but none have focused specifically on MedRec and its constituent tasks and subtasks. As medication errors remain a leading cause of preventable patient harm, mapping the MedRec processes to which AI has been applied is an important step toward understanding its role in facilitating MedRec automation. This is the first review to examine the use of AI for the automation of MedRec processes broken down by specific tasks and subtasks.

The objective of this scoping review was to map the existing literature on the use of AI for MedRec tasks and subtasks, with a focus on assessing the types of functions automated by the models or tools.

## Methods

We conducted this scoping review following guidance from the Joanna Briggs Institute [34]. This review is reported in accordance with the PRISMA-ScR (Preferred Reporting Items for Systematic Reviews and Meta-Analyses extension for Scoping Reviews; Checklist 1) [35].

### Eligibility Criteria

Following our protocol [36], eligible studies described applications of AI for a task related to MedRec. A previous review of electronic tools that support MedRec described MedRec as consisting of three core tasks: (1) creation of a BPMH, (2) identification of discrepancies among medication lists, and (3) resolution of medication discrepancies [28]. On the basis of these criteria, we included studies that described the development or evaluation of an AI model or AI-based tool that facilitated any of these tasks or a subtask associated with any of these tasks. To maintain relevance, we ensured that the primary objectives of the included studies aligned with MedRec tasks or subtasks.

Previous scoping reviews have mapped the use of AI or machine learning (ML) for purposes related to medication adherence and ADEs [31,37]. Thus, to avoid duplicating existing work, we excluded studies focusing on adherence, side effects, adverse drug reactions, or allergies, narrowing our review to the core tasks of MedRec. Similarly, we excluded studies restricted to specific medication classes to ensure broader applicability. We also excluded models or tools that were solely rule-based, instead focusing on those that were ML-based or incorporated both ML and rule-based methods.

We included studies involving participants of any population, as well as those focusing on nonhuman elements such as medication lists. We excluded studies dealing specifically with animals or veterinary medicine. We considered all health care settings (eg, inpatient, outpatient, and primary care). We included journal articles and certain types of gray literature, namely, conference papers and preprints, but excluded abstract-only reports. We considered structured reviews for inclusion but excluded other secondary literature.

### Information Sources, Search Strategy, and Study Selection

We undertook an initial preliminary search of MEDLINE (PubMed) to identify relevant articles. We used text words used in the titles and abstracts of these articles, as well as index terms used to describe them, to develop the full search strategy (Multimedia Appendix 1), which we adapted for each database. For the AI or ML component of our search, we adapted the AI- or ML-relevant terms from the search strategy used by Syrowatka et al [31] in their review of AI for ADE applications. The databases we searched were MEDLINE (Ovid), Embase (Ovid), Web of Science Core Collection, IEEE Xplore, and Compendex (Engineering Village). We searched the databases from inception to June 10, 2024, without any restrictions. In accordance with our eligibility criteria, we used a filter to exclude records indexed as involving animals and not humans. We also performed backward citation searching based on the articles included at the full-text stage using Citationchaser [38]. Due to resource constraints, we only considered English-language reports. Additional search details are presented in the PRISMA-S (Preferred Reporting Items for Systematic Reviews and Meta-Analyses literature search extension) [39] checklist (Checklist 2).

After completing the search, we uploaded all records retrieved into Rayyan (Qatar Computing Research Institute). One reviewer manually inspected all records flagged by Rayyan as potential duplicates for deduplication. Following deduplication, at least 2 independent reviewers performed title and abstract screening for each record. Where screeners disagreed about study inclusion, they discussed the records to reach consensus, consulting an additional reviewer if necessary. We retrieved the full texts of potentially relevant records and assessed them in the same manner.

### Data Extraction

We abstracted data using an extraction tool that we had piloted with 20% of the included studies (Multimedia Appendix 2 [30,40-132]). At least 2 independent reviewers extracted data for each study. Where discrepancies in data extraction arose, the extractors resolved them through discussion to reach consensus, consulting a third reviewer when consensus could not be achieved. We extracted information such as primary objectives, data sources, participant characteristics, training and evaluation set sizes, MedRec tasks and subtasks facilitated by the model or tool, ML methods used, and performance metrics for the best-performing model.

### Data Analysis

**Figure 1.**
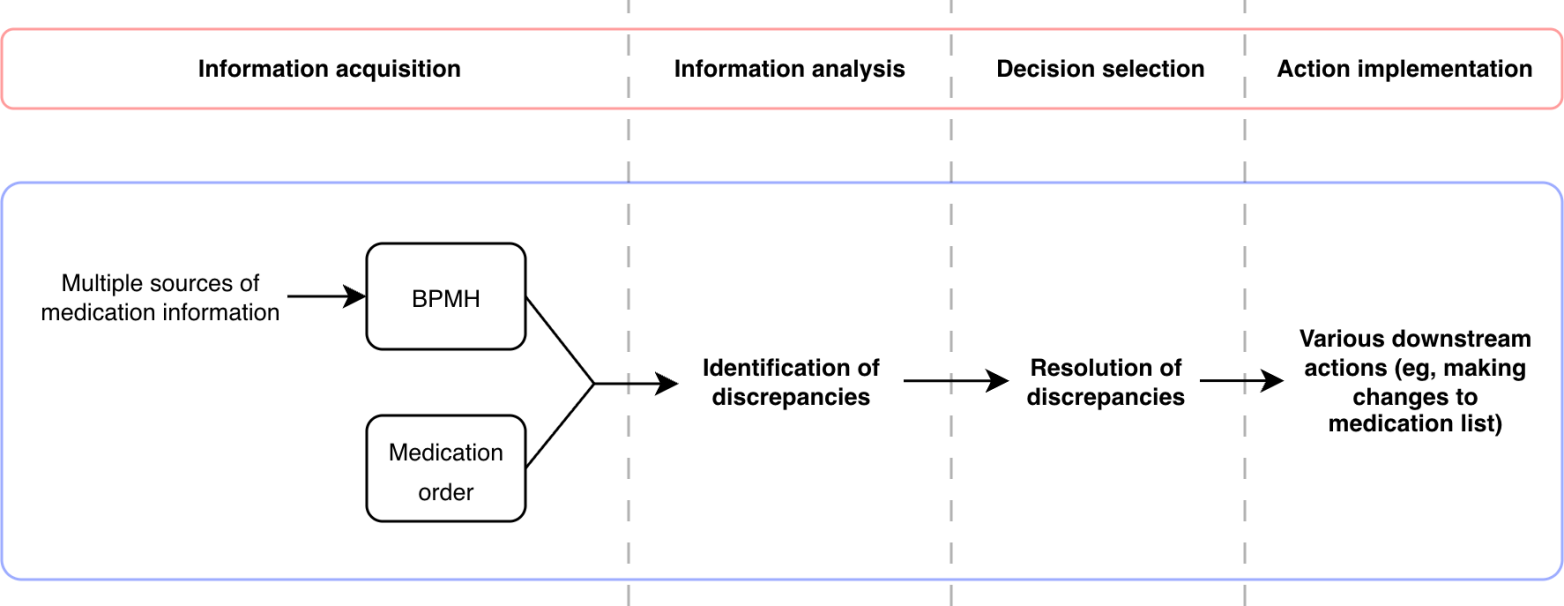
Stages of human information processing and corresponding medication reconciliation (MedRec) tasks (in an inpatient admission context) outlined by Marien et al [28]. BPMH: best possible medication history.

Previous research has explored the concept of automation from various perspectives. Our analytic approach was guided by the 4-stage model of human information processing by Parasuraman et al [133], which outlines system functions that can be automated, including information acquisition, information analysis, decision selection, and action implementation. We mapped the 3 core MedRec tasks of gathering medication data for the creation of a BPMH, identifying discrepancies, and resolving discrepancies (ie, evaluating discrepancies to determine actions) onto the first 3 stages of this model: information acquisition, information analysis, and decision selection, respectively (Figure 1 [28]). The fourth stage, action implementation, extends beyond the scope of core MedRec tasks as defined by Marien et al [28] but may include updating medication lists, communicating changes to the care team, or educating patients. This mapping approach provided a structured lens for assessing how AI-enabled automation could influence MedRec workflows.

## Results

### Review Findings

**Figure 2.**
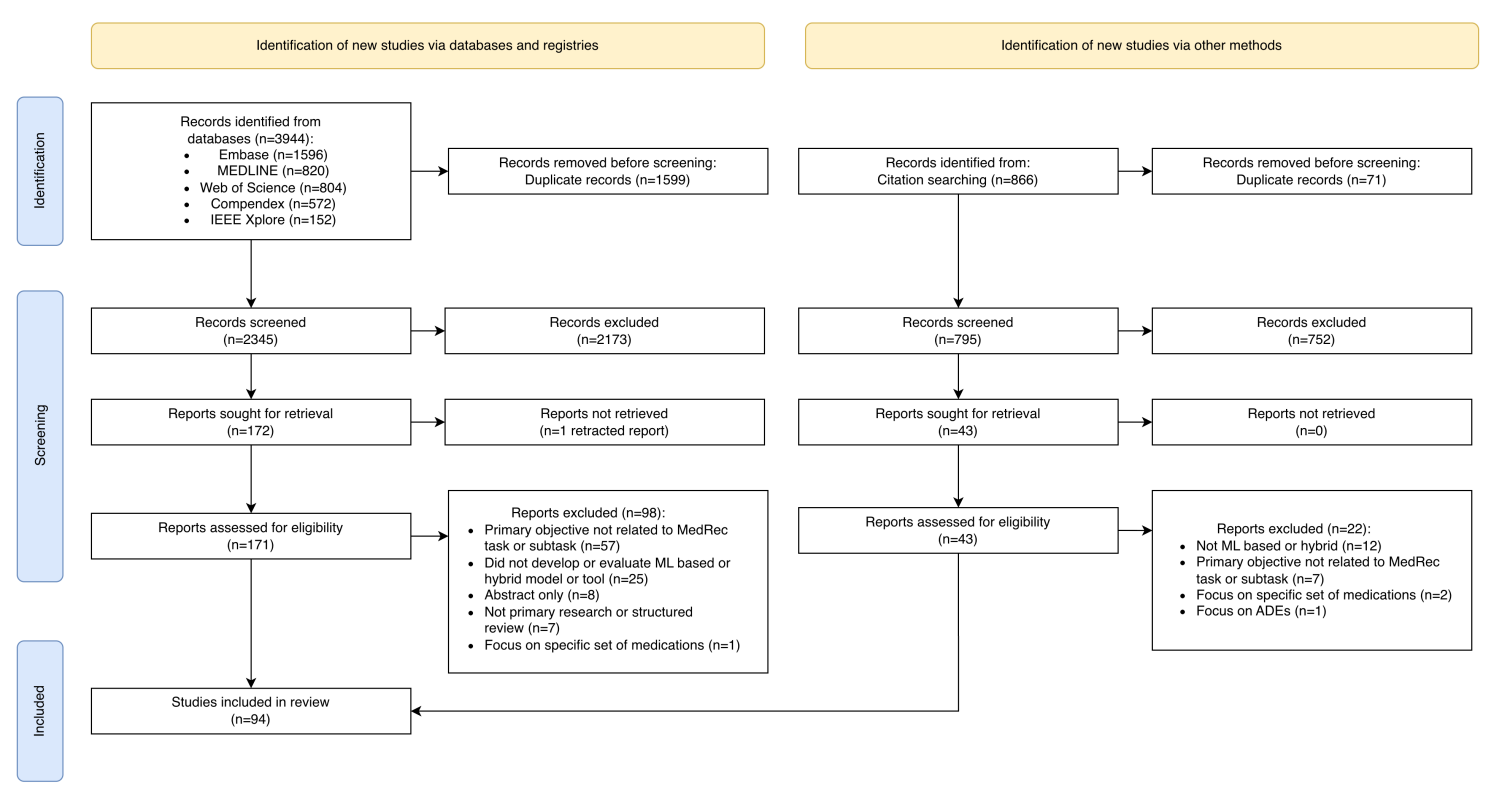
PRISMA (Preferred Reporting Items for Systematic Reviews and Meta-Analyses) flow diagram showing the study selection process. ADE: adverse drug event; MedRec: medication reconciliation; ML: machine learning.

We identified 2345 records through database searching and an additional 795 unique records from citation searching, ultimately including 94 studies (Figure 2).

We found that the included studies were largely restricted to 1 MedRec subtask, primarily using clinical notes in the electronic health record (EHR), although they used a variety of ML methods.

#### MedRec Tasks and Subtasks

**Figure 3.**
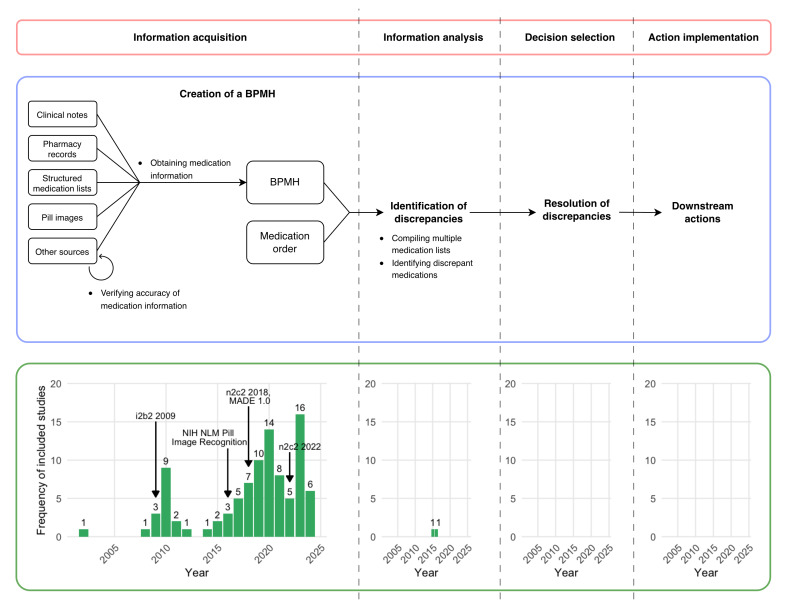
Conceptual mapping of medication reconciliation (MedRec) tasks and subtasks identified in this review onto the stages of human information processing and frequency of included studies by year. Subtasks are listed as bullet points. BPMH: best possible medication history; NIH: National Institutes of Health; NLM: National Library of Medicine.

All the included studies (94/94, 100%) focused on subtasks related to the creation of a BPMH, whereas 2.1% (2/94) also addressed the identification of discrepancies (Table 1). In terms of BPMH creation subtasks, most studies (92/94, 97.9%) extracted medication information from textual or visual sources [30,40-130]. Only 2.1% (2/94) of the studies focused on verifying the accuracy of already compiled medication information, and both of these studies leveraged a collaborative filtering approach to identify potential omissions from pharmacy medication lists [131,132]. Of the 2 studies that addressed the identification of discrepancies, both identified medications that were consistent as well as inconsistent between medication lists. One study used a conditional random field (CRF) model to extract medication entities from free-text clinical notes and compared them to medications extracted from structured discharge prescriptions using string matching, term co-occurrence, and drug synonyms, labeling medications from the clinical notes as “matched” or “discrepant” [80]. The other study developed a web-based application that retrieved medication lists from the EHR, allowing patients to manually confirm the extracted medications and add missing medications. Differences between the AI-extracted medications and patient-entered medications were flagged by the application [84]. No studies addressed the resolution of discrepancies between medication lists. As such, the highest stage of information processing automation achieved by the included models and tools was information analysis, with most studies (92/94, 97.9%) only automating information acquisition; none automated decision selection (Figure 3).

**Table 1.** Characteristics of the included studies (N=94).

Characteristic	Studies, n (%)
Year of publication	
2001‐2010	14 (14.9)
2011‐2020	45 (47.9)
2021‐2024	35 (37.2)
Region	
Africa	1 (1.1)
Asia	19 (20.2)
Australia	4 (4.4)
Europe	15 (16)
North America	55 (60.4)
Medication reconciliation task^a^	
Creation of a BPMH^b^	94 (100)
Identification of discrepancies	2 (2.1)
Resolution of discrepancies	0 (0)
ML^c^ method^a^	
Conditional random field	36 (38.3)
Recurrent neural network	34 (36.2)
Convolutional neural network	31 (33)
Transformer	25 (26.6)
Support vector machine	20 (21.3)
Maximum entropy	8 (8.5)
Random forest	6 (6.4)
K-nearest neighbor	6 (6.4)
Naive Bayes	4 (4.3)
K-means clustering	4 (4.3)
Other	21 (22.3)
Data source^a^	
Clinical note in EHR^d^	67 (71.3)
User-generated image	21 (22.3)
Pharmacy records	3 (3.2)
Patient-physician conversation transcript	2 (2.1)
Structured prescription list in the EHR	1 (1.1)
User-generated note	1 (1.1)
User-generated structured data	1 (1.1)
Medical internet forum	1 (1.1)
Data collection^a^	
Author-collected data	33 (35.1)
i2b2 2009 dataset	19 (20.2)
n2c2 2018 dataset	16 (17)
n2c2 2022 dataset	12 (12.8)
MADE 1.0 2018 dataset	7 (7.4)
NIH^e^ NLM^f^ Pill Image Recognition dataset	4 (4.3)
Other	19 (20.2)
Stage of implementation	
Model development, retrospective	93 (98.9)
Usability study	1 (1.1)

a Totals greater than 100%.

b BPMH: best possible medication history.

c ML: machine learning.

d EHR: electronic health record.

e NIH: National Institutes of Health.

f NLM: National Library of Medicine.

#### ML Methods

**Figure 4.**
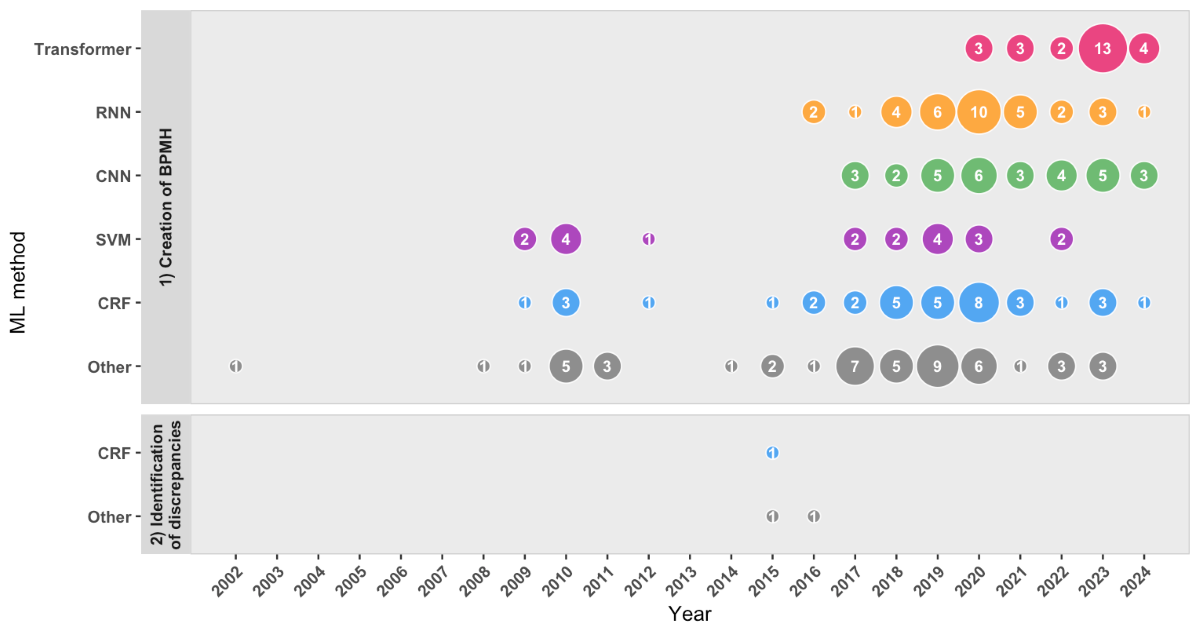
Evidence gap map illustrating the frequency of machine learning (ML) methods applied to medication reconciliation tasks by publication year. BPMH: best possible medication history; CNN: convolutional neural network; CRF: conditional random field; RNN: recurrent neural network; SVM: support vector machine.

The included studies used a variety of ML methods, with many using multiple methods (Figure 4). The most commonly used methods included CRFs (36/94, 38.3%) [30,41,43-45,48,52,55,57,67,68,70,71,73,75,78-81,94,95,99,100,103,109,115-117,119,123,126,127], recurrent neural networks (34/94, 36.2%) [30,40,41,45,48-52,59,61,67,68,70-74,76,78,81,85,91,92,94,95,101,103,109,119,123,125-127], convolutional neural networks (31/94, 33%) [40,42,45,49,52,59,64,72,74,76,77,81,82,87,90,92,95-97,101,102,108,112,114,119,120,122-124,127,130], transformers (25/94, 26.6%) [30,46,47,57,60,74,75,85-87,92-95,101,103-107,113,118,119,121,128], and support vector machines (20/94, 21.3%) [53,55,56,59,65,66,73,79,83,86,91,99,100,111,115,123,125-127,130]. Studies using free-text data (eg, notes in the EHR) typically used CRFs, recurrent neural networks, support vector machines, and transformers, whereas image-based studies mainly used convolutional neural networks. Owing to heterogeneity in task definitions (eg, differences in information extraction objectives and input modalities), performance metrics across studies were not directly comparable.

Among the studies that used free-text data sources (72/94, 76.6%), there was variation in the granularity of information extraction. Most of these studies (57/72, 79.2%) used ML-based named entity recognition techniques to extract a variety of medication-related elements, such as dosage, route of administration, frequency, and indication [30,41,43-53,55,57,58,60,61,63,67-71,73-75,78-81,85,91-95,99,100,103,104,106-109,113,115-119,121,123,125-128]. A smaller proportion of these studies (28/72, 38.9%) extended their approach to include relation extraction, identifying associations between these elements and specific medications (eg, linking a particular dosage to a specific medication) [30,41,43-45,47,50-52,63,73,78,79,81,87,91,93,99,100,106,107,115,116,123,125-128]. An even more limited subset (9/72, 12.5%) focused on identifying medication changes (eg, discontinuation or dosage adjustment) and the context of such changes (eg, the temporality of the change and the person responsible for the change) [46,47,57,86,103,104,106,118,121]. These studies typically assessed performance using standard evaluation metrics for natural language processing information extraction tasks, including precision, recall, and *F*_1_-score.

Studies that used image data (21/94, 22.3%) were primarily focused on medication identification. These studies frequently analyzed images of pills [42,77,82,96,97,102,112,114,120,122,124,129,130] or blister packs [66] to identify medications or generate a ranked list of possible medications. Others applied optical character recognition to pill bottle images or handwritten prescriptions and used natural language processing methods to extract medication information [59,76,101]. Most pill identification studies (10/13, 76.9%) used single-pill images, although a few (3/13, 23.1%) addressed multi-pill image identification [77,96,97]. No studies using pill images explicitly reported using patient-generated images, although many (8/13, 61.5%) incorporated intentional variations in lighting, zooming, and background, among other aspects, to simulate photos taken in real-world conditions [77,82,97,112,120,122,124,130]. The image-based studies often used more general accuracy metrics.

#### Data Sources and Collection

Most included studies (67/94, 71.3%) used clinical notes in the EHR [30,41,43-54,56,57,60-63,65,67,68,70,71,73-75,78-81,83-89,91,93-95,98-100,103-106,109-111,113,115-119,121,123,125-128], and a significant proportion of studies (21/94, 22.3%) used images [40,42,59,64,66,72,76,77,82,90,96,97,101,102,112,114,120,122,124,129,130]. In most studies using images, the images featured pills (13/21, 61.9%), although 38.1% (8/21) of the studies used images of other medication-related items (eg, medication bottles and handwritten prescriptions) [40,59,64,66,72,76,90,101]. A few studies used other sources, such as pharmacy records (3/94, 3.2%) [92,131,132], patient-physician conversation transcripts (2/94, 2.1%) [58,107], structured prescription lists in the EHR (1/94, 1.1%) [80], user-generated structured data (1/94, 1.1%) [84], user-generated notes (1/94, 1.1%) [108], or a medical internet forum (1/94, 1.1%) [48].

Although many studies (33/94, 35.1%) used author-collected data [40,42,56,58,59,61,64-66,80,83,84,90,96-99,101,107,108,111-114,124,129,131,132], most studies used publicly available data from various benchmarking shared tasks, including i2b2 2009 (19/94, 20.2%) [48,53-55,61-63,74,79,88,89,93,95,100,110,115-117,119], n2c2 2018 (16/94, 17%) [30,41,45,49,52,71,73,85,87,91,93-95,123,127,128], n2c2 2022 (12/94, 12.8%) [46,47,57,60,61,75,103-106,118,121], MADE 1.0 2018 (7/94, 7.4%) [43,44,50,51,81,125,126], and the National Institutes of Health National Library of Medicine Pill Image Recognition (4/94, 4.3%) datasets [77,120,122,130]. The i2b2 2009 and n2c2 2018 datasets consisted solely of discharge summaries [100,134], whereas the n2c2 2022 and MADE 1.0 datasets were not restricted to a single note type [135,136]. The n2c2 2018 and 2022 datasets were annotated by at least 2 independent expert annotators per note. While MADE 1.0 also used 2 annotators, they were not independent. In contrast, the i2b2 2009 dataset was released without annotations, requiring researchers to perform their own labeling. The National Library of Medicine Pill Image Recognition dataset includes both standardized, high-quality pill images and more variable consumer-quality images to reflect real-world user-generated image conditions [137].

#### Research Maturity

Most of the included studies (93/94, 98.9%) were limited to model development, with only 1.1% (1/94) of the studies advancing to a more mature stage of implementation [84].

## Discussion

### Principal Findings

#### Overview

This scoping review aimed to characterize how researchers have used AI to facilitate MedRec tasks and subtasks. Notably, all the included studies (94/94, 100%) addressed subtasks related to the creation of a BPMH, but only a handful (2/94, 2.1%) developed models or tools that facilitated discrepancy identification, and none facilitated discrepancy resolution. While there is growing interest in applying AI to support MedRec tasks, most research remains focused on demonstrating technical feasibility rather than evaluating clinical integration or real-world impact. As such, efforts to automate MedRec tasks using AI are still preliminary, with current work largely focused on a limited set of subtasks, whereas other subtasks remain unexamined.

#### Creation of a BPMH

The creation of a BPMH is a critical component of MedRec. One study of inpatient MedRec found that most unintentional medication discrepancies that had the potential to cause harm were the result of medication history errors [138], highlighting the importance of this task. In addition, BPMH creation can be time-consuming [139], emphasizing the value of automating aspects of this process.

Although all studies included in this review (94/94, 100%) developed models or tools that focused on subtasks of BPMH creation—such as medication extraction or verification—only 1.1% (1/94) developed a method that could potentially be applied to automate BPMH creation in an end-to-end manner [80]. This gap suggests that the end-to-end automation of MedRec tasks is limited not only by the state of AI but also by infrastructural barriers such as limitations to the quick exchange of medication data between EHRs and pharmacies or data incompleteness [140]. Previous work examining barriers to effective MedRec has identified the inability to access reliable medication information as a recurring challenge as users often lack access to records from external settings and the information present in EHRs may be incomplete or outdated [141-143]. These challenges may explain why so few studies (1/94, 1.1%) had progressed beyond model development to more advanced stages of implementation. Given that many models target information acquisition tasks, future research should examine what information is available to clinicians at the point of care, how these tools can augment that information, and the extent to which their use reduces cognitive and operational burden. A well-integrated medication repository that consolidates comprehensive drug histories across care settings could mitigate infrastructural challenges by improving data completeness and interoperability [144]. While such systems are not yet widely adopted or fully integrated across care settings, their expansion and improvement represent an important opportunity for future MedRec work. Efforts to update and make efficient use of health care infrastructure are key to facilitating the research, development, and implementation of tools that can scale the automation of MedRec.

The BPMH subtasks that the included studies addressed were relatively broad in scope (ie, collecting medication information from various sources and verifying the accuracy of this information). While BPMH creation is central to MedRec, its subtasks overlap functionally with other medication-related processes. This functional overlap highlights the potential for cross-task model reuse and suggests that automation efforts need not be strictly siloed within this first MedRec stage. Many of these models have broader applications beyond MedRec, including pharmacovigilance, adherence monitoring, and medication review (ie, evaluation and optimization of a patient’s medications [6]). More broadly, automating the creation of a complete medication list may enable downstream analytical use cases such as risk stratification (eg, age-related medication risks and fall risk from sedating medications), detection of potentially inappropriate prescribing or polypharmacy, and identification of deprescribing opportunities [145-147]. This broader relevance may partially explain the sustained research interest in developing these methods, particularly as public benchmarking initiatives such as the n2c2 2018 shared task on ADE and medication extraction [134] have incentivized the development of tools that support medication data processing across use cases.

The extent to which the included studies used data sources that reflect actual medication use is unclear, representing a key limitation in current BPMH automation research. The prevalence of patient nonadherence reported in the literature is often high [148], highlighting the importance of using sources of medication information that reflect actual use when developing a BPMH. The n2c2 2022 shared task on contextualized medication event extraction included identification of the “actor” responsible for medication changes (including patient-initiated changes) [135], suggesting that clinical notes may contain discernible indicators of actual use. Other data sources that may provide insights into actual medication use are patient-physician and patient-nurse conversation transcripts. As with clinical notes, such tools would require contextualized approaches to distinguish between mentions of prescribed regimens and actual use. Similarly, although pill image–based models could provide a closer approximation of actual use than health care professional-documented sources, studies have yet to validate them on patient-submitted images. Future research should focus on incorporating data sources that reflect actual medication use (eg, patient self-reported data) to improve the accuracy and completeness of the BPMH.

Some tools developed may also have relevance in virtual or remote care contexts. For instance, image-based methods for medication identification could be integrated into virtual care video visits to facilitate the creation of a BPMH (eg, by acting as an additional method to verify the medications taken by patients). This functionality could serve as a supplemental verification method, which may be useful for patients with communication barriers or low health literacy or managing complex medication regimens. As health care systems continue to expand telehealth offerings, these tools may become increasingly relevant for extending MedRec capabilities outside of traditional clinical environments.

#### Identification of Discrepancies

Beyond gathering medication data to create a BPMH, later MedRec tasks often require tailored approaches to identify and resolve discrepancies. This review found one study that compared medications from clinical notes and discharge prescriptions and another that flagged discrepancies between AI-extracted and patient-entered medication lists. Only one of these studies incorporated patient-entered data as a source of medication information, reflecting actual medication use [84]. The other study that supported this task compared structured discharge prescription lists and medications extracted from free-text clinical notes [80], although it is unclear whether such notes reflect prescribed regimens, actual use, or both. The limited adoption of AI for this task may be partially related to the existence of non–AI-based electronic MedRec tools that facilitate discrepancy identification. For example, the Twinlist MedRec tool uses rule-based processing to detect discrepancies, using visual elements such as spatial grouping and highlighting to guide users in addressing these discrepancies [22]. Other tools have adopted similar strategies [149,150]. However, AI models hold promise for automating information analysis more comprehensively by enabling more robust discrepancy identification (eg, in cases in which medication information is not standardized in structure) or handling more complex medication regimens (eg, dose tapering). Given that existing tools for discrepancy identification may already be addressed in some settings, AI and health system researchers should engage with clinicians to identify what MedRec tasks or subtasks would benefit most from automation, ensuring that the tools meaningfully complement clinical workflows.

#### Resolution of Discrepancies

Discrepancy resolution represents a significant opportunity for AI innovation in MedRec as it requires interpreting and integrating contextual information that current tools do not address. Inconsistent resolution practices may contribute to the lack of clear safety benefits from MedRec, highlighting the need for standardized approaches to discrepancy resolution [151]. This process often involves determining whether a discrepancy is intentional or unintentional, a subtask that may require additional details such as the timing of a medication change. The recent n2c2 2022 shared task on contextualized medication event extraction suggests that these considerations are receiving increasing attention [135]. For example, in addition to helping discern actual medication use from clinical notes, automated contextualized extraction could facilitate the identification of health care professional-initiated medication changes as well as the nature of such changes (eg, stopping a medication), which may aid decision-making in light of discrepancies. Still, curating datasets to train models for discrepancy resolution may be particularly challenging, requiring granular annotations of clinical intent that are rarely captured in real-world records. Furthermore, other organizational barriers to MedRec, such as ambiguity in roles and lack of standardized workflows [152,153], may be less amenable to technological solutions, emphasizing the importance of evaluating tools in real-world contexts where such factors are relevant.

The automation of decision selection in MedRec requires improved data collection practices and focused model development. To mitigate this gap without overburdening clinicians, one potential strategy is to focus structured data entry efforts on high-risk or high-variability medications where the clinical stakes are greatest, such as high-alert medications [140]. For instance, given the risks of severe hemorrhage associated with improper use of antithrombic medications [154], this medication group could be a key target for AI-assisted MedRec. Focusing on high-alert medication groups such as these would allow for more targeted model training while preserving overall workflow efficiency. Moreover, to automate this MedRec task effectively, future models must be capable of analyzing multiple data types—including clinical notes, structured orders, medication timelines, and even health care professional communication patterns—to infer intent and recommend appropriate actions. This multimodal challenge, which extends beyond collecting the correct information, remains an underexplored but essential area for advancing toward fully integrated, AI-assisted MedRec workflows.

### Limitations

There are limitations to this review. While the conceptual framework of MedRec tasks that we used provided a useful analytic structure, MedRec workflows vary across institutions. As such, the tasks and subtasks outlined in this review may not be universally applicable. Furthermore, although we framed the creation of a BPMH as the “information acquisition” step of MedRec, this process itself draws on medication information from multiple sources (eg, patient interview, pharmacy dispensing data, and EHR prescription lists). Hence, creating a BPMH also requires elements of information analysis and decision selection steps (eg, when sources used to create a BPMH are in conflict) such that the models and tools identified in this review are not necessarily restricted in their utility to just one MedRec task. In light of existing reviews, we excluded studies primarily focused on adherence, adverse drug reactions, side effects, or allergies; however, these aspects of medication use are relevant to comprehensive MedRec efforts. We direct readers to the work by Bohlmann et al [37] and Syrowatka et al [31] for syntheses of the literature on AI or ML for adherence and ADE applications, which include studies relevant to the creation of a BPMH.

Finally, our search did not capture literature published since mid-2024, and therefore, our findings would not reflect more recent approaches. There may also be some commercial MedRec systems that use AI but that have not been evaluated in a research context and that, therefore, our search would not have identified.

### Conclusions

AI holds potential for supporting MedRec by reducing manual workloads and improving efficiency. This work is the first to provide a comprehensive overview of the use of AI for MedRec, using an information processing framework to evaluate the degree of automation achieved across MedRec tasks. While previous reviews have explored the use of electronic tools for MedRec, none have focused on AI applications specifically. Our review highlights which MedRec tasks have received the most AI research effort and where gaps remain, providing valuable direction for future work that aims to advance the automation of MedRec. Current AI research in MedRec remains concentrated on a limited set of subtasks, primarily at the model development stage. To fully harness the potential of AI in MedRec, researchers and health care systems should approach implementation critically, emphasizing iterative evaluation and refinement. The aim is augmentation, not replacement. Rather than a simple transfer of responsibilities from human to machine, automation should be viewed as a dynamic process featuring ongoing user and AI tool interactions, as well as ongoing verification and refinement [155-157]. Researchers should balance refining AI-based models with the prevention of medication-related harms. Future research should prioritize developing models to automate discrepancy resolution, developing and evaluating AI-based MedRec tools, and addressing infrastructural barriers to broader implementation. Health systems need usable, well-integrated tools, as well as translational studies to assess their real-world effectiveness and safety. These tools will operate within complex sociotechnical systems, where success depends not only on model performance but also on usability, interoperability, and trust.

## Supplementary material

10.2196/86760 Multimedia Appendix 1 Full search strategies.

10.2196/86760 Multimedia Appendix 2 Extraction table.

10.2196/86760 Checklist 1 PRISMA-ScR checklist.

10.2196/86760 Checklist 2 PRISMA-S checklist.

10.2196/86760 Checklist 3 PRISMA 2020 for Abstracts checklist.

## Abbreviations

ADE: adverse drug event
AI: artificial intelligence
BPMH: best possible medication history
CRF: conditional random field
EHR: electronic health record
MedRec: medication reconciliation
ML: machine learning
PRISMA-S: Preferred Reporting Items for Systematic Reviews and Meta-Analyses literature search extension
PRISMA-ScR: Preferred Reporting Items for Systematic Reviews and Meta-Analyses extension for Scoping Reviews
