# Supplementary material for: AI-Based Automation for Medication Reconciliation: Scoping Review
Source: J Med Internet Res. 2026 May 11;28:e86760. doi: 10.2196/86760 (PMC13160534; doi:10.2196/86760)
Supplement: Multimedia Appendix 1 [file jmir-v28-e86760-s001.docx]

# Appendix B. Full Search Strategy

## MEDLINE

| 1 | (machine learning or artificial intelligence or AI).mp. or Artificial Intelligence/ or exp Machine Learning/ or exp Expert Systems/ or exp Pattern Recognition, Automated/ |
| --- | --- |
| 2 | (decision tree* or gradient boost* or random forest*).mp. |
| 3 | Natural Language Processing/ or (NLP or natural language processing).mp. |
| 4 | exp Neural Networks, Computer/ or (neural network* or deep learning).mp. |
| 5 | (pattern recognition or pattern identification or computational intelligence* or machine intelligence* or intelligent system* or computer reasoning or expert system*).mp. |
| 6 | (supervised learning or unsupervised learning).mp. |
| 7 | (perceptron* or connectionist* or adversarial network* or graph convolutional network* or transformer*).mp. |
| 8 | (support vector* or multiple kernel or multi kernel or multikernel or structured kernel or knn or rnn or nearest neighb* or kmeans or k-means or k means or ensemble learning or ensemble model* or transfer learning or reinforcement learning).mp. |
| 9 | (bayes* or fuzzy logic).mp. |
| 10 | or/1-9 [ML and AI] |
| 11 | ((pharmaco* or pharmaceutic* or drug* or medication* or medicine* or prescri* or discrepanc*) adj2 reconcil*).mp. |
| 12 | ((pharmaco* or pharmaceutic* or drug* or medication* or medicine* or prescri*) adj2 discrepanc*).mp. |
| 13 | ((pharmaco* or pharmaceutic* or medication* or medicine* or prescri*) adj2 list*).mp. |
| 14 | (((medication* or medicine* or prescri*) adj2 (history or histories)) not history of medicine).mp. |
| 15 | ((medication* or medicine* or prescri*) adj2 (extract* or retriev*)).mp. |
| 16 | ((medication* or medicine* or prescri* or pill*) adj2 identif*).mp. |
| 17 | ((medication* or medicine* or prescri*) adj2 change*).mp. |
| 18 | ((medication* or medicine* or prescri*) adj2 information).mp. |
| 19 | Medication Reconciliation/ |
| 20 | or/11-19 [MedRec] |
| 21 | 10 and 20 |
| 22 | exp animals/ not humans.sh. |
| 23 | 21 not 22 |

## Embase

| 1 | (machine learning or artificial intelligence or AI).mp. or exp artificial intelligence/ or exp expert system/ or exp machine learning/ or exp automated pattern recognition/ |
| --- | --- |
| 2 | (decision tree* or gradient boost* or random forest*).mp. |
| 3 | natural language processing/ or (NLP or natural language process*).mp. |
| 4 | (neural network* or deep learning).mp. |
| 5 | (pattern recognition or pattern identification or computational intelligence or machine intelligence or intelligent system* or computer reasoning or expert system*).mp. |
| 6 | (supervised learning or unsupervised learning).mp. |
| 7 | (perceptron* or connectionist* or adversarial network* or graph convolutional network* or transformer*).mp. |
| 8 | (support vector* or multiple kernel or multi kernel or multikernel or structured kernel or knn or rnn or nearest neighb* or kmeans or k-means or k means or ensemble learning or ensemble model* or transfer learning or reinforcement learning).mp. |
| 9 | (bayes* or fuzzy logic).mp. |
| 10 | or/1-9 [ML and AI] |
| 11 | ((pharmaco* or pharmaceutic* or drug* or medication* or medicine* or prescri* or discrepanc*) adj2 reconcil*).mp. |
| 12 | ((pharmaco* or pharmaceutic* or drug* or medication* or medicine* or prescri*) adj2 discrepanc*).mp. |
| 13 | ((pharmaco* or pharmaceutic* or medication* or medicine* or prescri*) adj2 list*).mp. |
| 14 | (((medication* or medicine* or prescri*) adj2 (history or histories)) not history of medicine).mp. |
| 15 | ((medication* or medicine* or prescri*) adj2 (extract* or retriev*)).mp. |
| 16 | ((medication* or medicine* or prescri* or pill*) adj2 identif*).mp. |
| 17 | ((medication* or medicine* or prescri*) adj2 change*).mp. |
| 18 | ((medication* or medicine* or prescri*) adj2 information).mp. |
| 19 | medication therapy management/ |
| 20 | or/11-19 [MedRec] |
| 21 | 10 and 20 |
| 22 | (exp animals/ or nonhuman/) not exp human/ |
| 23 | 21 not 22 |

## Compendex

(((pharmacotherapy NEAR/1 reconciliation) OR (pharmacologic NEAR/1 reconciliation) OR (pharmacologics NEAR/1 reconciliation) OR (pharmaceutical NEAR/1 reconciliation) OR (pharmaceuticals NEAR/1 reconciliation) OR (drug NEAR/1 reconciliation) OR (drugs NEAR/1 reconciliation) OR (medication NEAR/1 reconciliation) OR (medications NEAR/1 reconciliation) OR (medicine NEAR/1 reconciliation) OR (medicines NEAR/1 reconciliation) OR (prescription NEAR/1 reconciliation) OR (prescriptions NEAR/1 reconciliation) OR (prescribing NEAR/1 reconciliation) OR (discrepancy NEAR/1 reconciliation) OR (discrepancies NEAR/1 reconciliation) OR (pharmacotherapy NEAR/1 reconcile) OR (pharmacologic NEAR/1 reconcile) OR (pharmacologics NEAR/1 reconcile) OR (pharmaceutical NEAR/1 reconcile) OR (pharmaceuticals NEAR/1 reconcile) OR (drug NEAR/1 reconcile) OR (drugs NEAR/1 reconcile) OR (medication NEAR/1 reconcile) OR (medications NEAR/1 reconcile) OR (medicine NEAR/1 reconcile) OR (medicines NEAR/1 reconcile) OR (prescription NEAR/1 reconcile) OR (prescriptions NEAR/1 reconcile) OR (prescribing NEAR/1 reconcile) OR (discrepancy NEAR/1 reconcile) OR (discrepancies NEAR/1 reconcile) OR (pharmacotherapy NEAR/1 reconciling) OR (pharmacologic NEAR/1 reconciling) OR (pharmacologics NEAR/1 reconciling) OR (pharmaceutical NEAR/1 reconciling) OR (pharmaceuticals NEAR/1 reconciling) OR (drug NEAR/1 reconciling) OR (drugs NEAR/1 reconciling) OR (medication NEAR/1 reconciling) OR (medications NEAR/1 reconciling) OR (medicine NEAR/1 reconciling) OR (medicines NEAR/1 reconciling) OR (prescription NEAR/1 reconciling) OR (prescriptions NEAR/1 reconciling) OR (prescribing NEAR/1 reconciling) OR (discrepancy NEAR/1 reconciling) OR (discrepancies NEAR/1 reconciling) OR (pharmacotherapy NEAR/1 reconciled) OR (pharmacologic NEAR/1 reconciled) OR (pharmacologics NEAR/1 reconciled) OR (pharmaceutical NEAR/1 reconciled) OR (pharmaceuticals NEAR/1 reconciled) OR (drug NEAR/1 reconciled) OR (drugs NEAR/1 reconciled) OR (medication NEAR/1 reconciled) OR (medications NEAR/1 reconciled) OR (medicine NEAR/1 reconciled) OR (medicines NEAR/1 reconciled) OR (prescription NEAR/1 reconciled) OR (prescriptions NEAR/1 reconciled) OR (prescribing NEAR/1 reconciled) OR (discrepancy NEAR/1 reconciled) OR (discrepancies NEAR/1 reconciled) OR (pharmacotherapy NEAR/1 discrepancy) OR (pharmacologic NEAR/1 discrepancy) OR (pharmacologics NEAR/1 discrepancy) OR (pharmaceutical NEAR/1 discrepancy) OR (pharmaceuticals NEAR/1 discrepancy) OR (drug NEAR/1 discrepancy) OR (drugs NEAR/1 discrepancy) OR (medication NEAR/1 discrepancy) OR (medications NEAR/1 discrepancy) OR (medicine NEAR/1 discrepancy) OR (medicines NEAR/1 discrepancy) OR (prescription NEAR/1 discrepancy) OR (prescriptions NEAR/1 discrepancy) OR (prescribing NEAR/1 discrepancy) OR (pharmacotherapy NEAR/1 discrepancies) OR (pharmacologic NEAR/1 discrepancies) OR (pharmacologics NEAR/1 discrepancies) OR (pharmaceutical NEAR/1 discrepancies) OR (pharmaceuticals NEAR/1 discrepancies) OR (drug NEAR/1 discrepancies) OR (drugs NEAR/1 discrepancies) OR (medication NEAR/1 discrepancies) OR (medications NEAR/1 discrepancies) OR (medicine NEAR/1 discrepancies) OR (medicines NEAR/1 discrepancies) OR (prescription NEAR/1 discrepancies) OR (prescriptions NEAR/1 discrepancies) OR (prescribing NEAR/1 discrepancies) OR (pharmacotherapy NEAR/1 list) OR (pharmacologic NEAR/1 list) OR (pharmacologics NEAR/1 list) OR (pharmaceutical NEAR/1 list) OR (pharmaceuticals NEAR/1 list) OR (medication NEAR/1 list) OR (medications NEAR/1 list) OR (medicine NEAR/1 list) OR (medicines NEAR/1 list) OR (prescription NEAR/1 list) OR (prescriptions NEAR/1 list) OR (prescribing NEAR/1 list) OR (pharmacotherapy NEAR/1 lists) OR (pharmacologic NEAR/1 lists) OR (pharmacologics NEAR/1 lists) OR (pharmaceutical NEAR/1 lists) OR (pharmaceuticals NEAR/1 lists) OR (medication NEAR/1 lists) OR (medications NEAR/1 lists) OR (medicine NEAR/1 lists) OR (medicines NEAR/1 lists) OR (prescription NEAR/1 lists) OR (prescriptions NEAR/1 lists) OR (prescribing NEAR/1 lists) OR (((medication NEAR/1 history) OR (medications NEAR/1 history) OR (medicine NEAR/1 history) OR (medicines NEAR/1 history) OR (prescription NEAR/1 history) OR (prescriptions NEAR/1 history) OR (prescribing NEAR/1 history) OR (medication NEAR/1 histories) OR (medications NEAR/1 histories) OR (medicine NEAR/1 histories) OR (medicines NEAR/1 histories) OR (prescription NEAR/1 histories) OR (prescriptions NEAR/1 histories) OR (prescribing NEAR/1 histories)) NOT "history of medicine") OR (medication NEAR/1 extract) OR (medications NEAR/1 extract) OR (medicine NEAR/1 extract) OR (medicines NEAR/1 extract) OR (prescription NEAR/1 extract) OR (prescriptions NEAR/1 extract) OR (prescribing NEAR/1 extract) OR (medication NEAR/1 extraction) OR (medications NEAR/1 extraction) OR (medicine NEAR/1 extraction) OR (medicines NEAR/1 extraction) OR (prescription NEAR/1 extraction) OR (prescriptions NEAR/1 extraction) OR (prescribing NEAR/1 extraction) OR (medication NEAR/1 extracted) OR (medications NEAR/1 extracted) OR (medicine NEAR/1 extracted) OR (medicines NEAR/1 extracted) OR (prescription NEAR/1 extracted) OR (prescriptions NEAR/1 extracted) OR (prescribing NEAR/1 extracted) OR (medication NEAR/1 extracting) OR (medications NEAR/1 extracting) OR (medicine NEAR/1 extracting) OR (medicines NEAR/1 extracting) OR (prescription NEAR/1 extracting) OR (prescriptions NEAR/1 extracting) OR (prescribing NEAR/1 extracting) OR (medication NEAR/1 retrieve) OR (medications NEAR/1 retrieve) OR (medicine NEAR/1 retrieve) OR (medicines NEAR/1 retrieve) OR (prescription NEAR/1 retrieve) OR (prescriptions NEAR/1 retrieve) OR (prescribing NEAR/1 retrieve) OR (medication NEAR/1 retrieval) OR (medications NEAR/1 retrieval) OR (medicine NEAR/1 retrieval) OR (medicines NEAR/1 retrieval) OR (prescription NEAR/1 retrieval) OR (prescriptions NEAR/1 retrieval) OR (prescribing NEAR/1 retrieval) OR (medication NEAR/1 retrieving) OR (medications NEAR/1 retrieving) OR (medicine NEAR/1 retrieving) OR (medicines NEAR/1 retrieving) OR (prescription NEAR/1 retrieving) OR (prescriptions NEAR/1 retrieving) OR (prescribing NEAR/1 retrieving) OR (medication NEAR/1 retrieved) OR (medications NEAR/1 retrieved) OR (medicine NEAR/1 retrieved) OR (medicines NEAR/1 retrieved) OR (prescription NEAR/1 retrieved) OR (prescriptions NEAR/1 retrieved) OR (prescribing NEAR/1 retrieved) OR (medication NEAR/1 identify) OR (medications NEAR/1 identify) OR (medicine NEAR/1 identify) OR (medicines NEAR/1 identify) OR (prescription NEAR/1 identify) OR (prescriptions NEAR/1 identify) OR (prescribing NEAR/1 identify) OR (pill NEAR/1 identify) OR (pills NEAR/1 identify) OR (medication NEAR/1 identification) OR (medications NEAR/1 identification) OR (medicine NEAR/1 identification) OR (medicines NEAR/1 identification) OR (prescription NEAR/1 identification) OR (prescriptions NEAR/1 identification) OR (prescribing NEAR/1 identification) OR (pill NEAR/1 identification) OR (pills NEAR/1 identification) OR (medication NEAR/1 identified) OR (medications NEAR/1 identified) OR (medicine NEAR/1 identified) OR (medicines NEAR/1 identified) OR (prescription NEAR/1 identified) OR (prescriptions NEAR/1 identified) OR (prescribing NEAR/1 identified) OR (pill NEAR/1 identified) OR (pills NEAR/1 identified) OR (medication NEAR/1 identifying) OR (medications NEAR/1 identifying) OR (medicine NEAR/1 identifying) OR (medicines NEAR/1 identifying) OR (prescription NEAR/1 identifying) OR (prescriptions NEAR/1 identifying) OR (prescribing NEAR/1 identifying) OR (pill NEAR/1 identifying) OR (pills NEAR/1 identifying) OR (medication NEAR/1 change) OR (medications NEAR/1 change) OR (medicine NEAR/1 change) OR (medicines NEAR/1 change) OR (prescription NEAR/1 change) OR (prescriptions NEAR/1 change) OR (prescribing NEAR/1 change) OR (medication NEAR/1 changes) OR (medications NEAR/1 changes) OR (medicine NEAR/1 changes) OR (medicines NEAR/1 changes) OR (prescription NEAR/1 changes) OR (prescriptions NEAR/1 changes) OR (prescribing NEAR/1 changes) OR (medication NEAR/1 changed) OR (medications NEAR/1 changed) OR (medicine NEAR/1 changed) OR (medicines NEAR/1 changed) OR (prescription NEAR/1 changed) OR (prescriptions NEAR/1 changed) OR (prescribing NEAR/1 changed) OR (medication NEAR/1 information) OR (medications NEAR/1 information) OR (medicine NEAR/1 information) OR (medicines NEAR/1 information) OR (prescription NEAR/1 information) OR (prescriptions NEAR/1 information) OR (prescribing NEAR/1 information)) WN KY) AND ((("artificial intelligence" OR AI OR "machine intelligence" OR "computational intelligence" OR "machine learning" OR "intelligent system*" OR "computer reasoning" OR "pattern recognition" OR "pattern identification" OR "expert system*" OR "supervised learning" OR "unsupervised learning" OR "deep learning" OR "neural network*"OR "decision tree*" OR "random forest*" OR perceptron* OR connectionist* OR "gradient boost*" OR "adversarial network*" OR "graph convolutional network*" OR "support vector*" OR "multiple kernel" OR "multi kernel" OR multikernel OR "structured kernel" OR knn OR rnn OR "nearest neighb*" OR kmeans OR "k-means" OR "ensemble learning" OR "ensemble model" OR "transfer learning" OR "reinforcement learning" OR "natural language processing" OR "natural language processor" OR bayes* OR "fuzzy logic" OR transformer*) WN KY) OR ({Artificial intelligence} WN CV) OR ({Medical information systems} WN CV) OR ({Expert systems} WN CV))

## IEEE Xplore

("Abstract":"artificial intelligence" OR "Abstract":AI OR "Abstract":"machine intelligence" OR "Abstract":"computational intelligence" OR "Abstract":"machine learning" OR "Abstract":"expert system" OR "Abstract":"intelligent system" OR "Abstract":"computer reasoning" OR "Abstract":"pattern recognition" OR "Abstract":"pattern identification" OR "Abstract":"supervised learning" OR "Abstract":"unsupervised learning" OR "Abstract":"deep learning" OR "Abstract":"decision tree" OR "Abstract":"decision trees" OR "Abstract":"random forest" OR "Abstract":"random forests" OR "Abstract":"gradient boosting" OR "Abstract":perceptron OR "Abstract":connectionist OR "Abstract":"adversarial network" OR "Abstract":"graph convolutional network" OR "Abstract":"support vector" OR "Abstract":"multiple kernel" OR "Abstract":"multi kernel" OR "Abstract":multikernel OR "Abstract":"structured kernel" OR "Abstract":knn OR "Abstract":rnn OR "Abstract":"nearest neighbor" OR "Abstract":"nearest neighbors" OR "Abstract":kmeans OR "Abstract":"k-means" OR "Abstract":"ensemble learning" OR "Abstract":"ensemble model" OR "Abstract":"transfer learning" OR "Abstract":"reinforcement learning" OR "Abstract":"natural language processing" OR "Abstract":"natural language processor" OR "Abstract":bayes OR "Abstract":bayesian OR "Abstract":"fuzzy logic" OR "Abstract":transformer OR "Abstract":transformers OR "Abstract":"neural network" OR "Abstract":"neural networks") AND ((("Abstract":pharmacotherapy OR "Abstract":pharmacologic OR "Abstract":pharmacologics OR "Abstract":pharmaceutic OR "Abstract":pharmaceutics OR "Abstract":pharmaceutical OR "Abstract":pharmaceuticals OR "Abstract":drug OR "Abstract":drugs OR "Abstract":medicine OR "Abstract":medicines OR "Abstract":medication OR "Abstract":medications OR "Abstract":prescribing OR "Abstract":prescription OR "Abstract":prescriptions OR "Abstract":discrepancy OR "Abstract":discrepancies) NEAR/2 ("Abstract":reconcil*)) OR (("Abstract":pharmacotherapy OR "Abstract":pharmacologic OR "Abstract":pharmacologics OR "Abstract":pharmaceutic OR "Abstract":pharmaceutics OR "Abstract":pharmaceutical OR "Abstract":pharmaceuticals OR "Abstract":drug OR "Abstract":drugs OR "Abstract":medicine OR "Abstract":medicines OR "Abstract":medication OR "Abstract":medications OR "Abstract":prescribing OR "Abstract":prescription OR "Abstract":prescriptions) NEAR/2 ("Abstract":discrepancy OR "Abstract":discrepancies OR "Abstract":list OR "Abstract":lists)) OR (("Abstract":medication OR "Abstract":medications OR "Abstract":medicine OR "Abstract":medicines OR "Abstract":prescribing OR "Abstract":prescription OR "Abstract":prescriptions) NEAR/2 ("Abstract":histor* OR "Abstract":extract* OR "Abstract":retriev* OR "Abstract":information OR "Abstract":change*)) OR (("Abstract":medication OR "Abstract":medications OR "Abstract":medicine OR "Abstract":medicines OR "Abstract":prescribing OR "Abstract":prescription OR "Abstract":prescriptions OR "Abstract":pill OR "Abstract":pills) NEAR/2 ("Abstract":identif*)))

## Web of Science

| # | Search Query |
| --- | --- |
| 1 | AB=(("artificial intelligence" OR "machine intelligence" OR AI OR "computational intelligence" OR "machine learning" OR "expert system*" OR "pattern recognition" OR "pattern identification" OR "intelligent system*" OR "computer reasoning" OR "supervised learning" OR "unsupervised learning" OR "deep learning" OR "neural network*" OR "decision tree*" OR "random forest*" OR perceptron* OR connectionist* OR "gradient boost*" OR "adversarial network*" OR "graph convolutional network*" OR "support vector*" OR "multiple kernel" OR "multi kernel" OR multikernel OR "structured kernel" OR knn OR rnn OR "nearest neighb*" OR kmeans OR "k-means" OR "ensemble learning" OR "ensemble model" OR "transfer learning" OR "fuzzy logic" OR "reinforcement learning" OR "natural language processing" OR "natural language processor" OR bayes* OR transformer*) AND (((pharmaco* OR pharmaceutic* OR drug* OR medication* OR medicine* OR prescri* OR discrepanc*) NEAR/1 reconcil*) OR ((pharmaco* OR pharmaceutic* OR drug* OR medication* OR medicine* OR prescri*) NEAR/1 discrepanc*) OR ((pharmaco* OR pharmaceutic* OR medication* OR medicine* OR prescri*) NEAR/1 list*) OR (((medication* OR medicine* OR prescri*) NEAR/1 (history or histories)) NOT “history of medicine”) OR ((medication* OR medicine* OR prescri*) NEAR/1 (extract* OR retriev*)) OR ((medication* OR medicine* OR prescri* OR pill*) NEAR/1 identif*) OR ((medication* OR medicine* OR prescri*) NEAR/1 change*) OR ((medication* OR medicine* OR prescri*) NEAR/1 information))) |
| 2 | TI=(("artificial intelligence" OR "machine intelligence" OR AI OR "computational intelligence" OR "machine learning" OR "expert system*" OR "pattern recognition" OR "pattern identification" OR "intelligent system*" OR "computer reasoning" OR "supervised learning" OR "unsupervised learning" OR "deep learning" OR "neural network*" OR "decision tree*" OR "random forest*" OR perceptron* OR connectionist* OR "gradient boost*" OR "adversarial network*" OR "graph convolutional network*" OR "support vector*" OR "multiple kernel" OR "multi kernel" OR multikernel OR "structured kernel" OR knn OR rnn OR "nearest neighb*" OR kmeans OR "k-means" OR "ensemble learning" OR "ensemble model" OR "transfer learning" OR "fuzzy logic" OR "reinforcement learning" OR "natural language processing" OR "natural language processor" OR bayes* OR transformer*) AND (((pharmaco* OR pharmaceutic* OR drug* OR medication* OR medicine* OR prescri* OR discrepanc*) NEAR/1 reconcil*) OR ((pharmaco* OR pharmaceutic* OR drug* OR medication* OR medicine* OR prescri*) NEAR/1 discrepanc*) OR ((pharmaco* OR pharmaceutic* OR medication* OR medicine* OR prescri*) NEAR/1 list*) OR (((medication* OR medicine* OR prescri*) NEAR/1 (history or histories)) NOT “history of medicine”) OR ((medication* OR medicine* OR prescri*) NEAR/1 (extract* OR retriev*)) OR ((medication* OR medicine* OR prescri* OR pill*) NEAR/1 identif*) OR ((medication* OR medicine* OR prescri*) NEAR/1 change*) OR ((medication* OR medicine* OR prescri*) NEAR/1 information))) |
| 3 | #1 OR #2 |
