# Supplementary material for: AI-Based Automation for Medication Reconciliation: Scoping Review
Source: J Med Internet Res. 2026 May 11;28:e86760. doi: 10.2196/86760 (PMC13160534; doi:10.2196/86760)
Supplement: Checklist 2 [file jmir-v28-e86760-s004.pdf]

PRISMA-S Checklist

Section/topic	#	Checklist item	Location(s) Reported
**INFORMATION SOURCES AND METHODS**			
Database name	1	Name each individual database searched, stating the platform for each.	p. 4
Multi-database searching	2	If databases were searched simultaneously on a single platform, state the name of the platform, listing all of the databases searched.	NA[i]
Study registries	3	List any study registries searched.	NA[ii]
Online resources and browsing	4	Describe any online or print source purposefully searched or browsed (e.g., tables of contents, print conference proceedings, web sites), and how this was done.	NA[iii]
Citation searching	5	Indicate whether cited references or citing references were examined, and describe any methods used for locating cited/citing references (e.g., browsing reference lists, using a citation index, setting up email alerts for references citing included studies).	p. 5
Contacts	6	Indicate whether additional studies or data were sought by contacting authors, experts, manufacturers, or others.	NA[iv]
Other methods	7	Describe any additional information sources or search methods used.	NA[iv]
**SEARCH STRATEGIES**			
Full search strategies	8	Include the search strategies for each database and information source, copied and pasted exactly as run.	Appendix 2
Limits and restrictions	9	Specify that no limits were used, or describe any limits or restrictions applied to a search (e.g., date or time period, language, study design) and provide justification for their use.	p. 4-5
Search filters	10	Indicate whether published search filters were used (as originally designed or modified), and if so, cite the filter(s) used.	p. 5
Prior work	11	Indicate when search strategies from other literature reviews were adapted or reused for a substantive part or all of the search, citing the previous review(s).	p. 4
Updates	12	Report the methods used to update the search(es) (e.g., rerunning searches, email alerts).	NA[v]

Dates of searches	13	For each search strategy, provide the date when the last search occurred.	p. 4
**PEER REVIEW**			
Peer review	14	Describe any search peer review process.	NA[vi]
**MANAGING RECORDS**			
Total Records	15	Document the total number of records identified from each database and other information sources.	p. 6[vii]
Deduplication	16	Describe the processes and any software used to deduplicate records from multiple database searches and other information sources.	p. 5

PRISMA-S: An Extension to the PRISMA Statement for Reporting Literature Searches in Systematic Reviews
Rethlefsen ML, Kirtley S, Waffenschmidt S, Ayala AP, Moher D, Page MJ, Koffel JB, PRISMA-S Group.
Last updated February 27, 2020.

[i] Searches were performed separately for each database.
[ii] We did not search any study registries as preliminary searches suggested the literature was predominantly at a discovery or feasibility stage and unlikely to include registered trials.
[iii] We did not search online or print sources, though some of the databases we searched index conference papers and/or preprints (Embase, Web of Science, IEEE Xplore, Compendex).
[iv] We did not contact authors or other parties for additional studies, nor did we employ other search methods such as personal file searches or database-specific search tools (e.g., ‘related articles’ features), as we considered the breadth of our database search and citation searching sufficient to capture the relevant literature, and wished to ensure the reproducibility of the search strategy.
[v] We did not update the search.
[vi] Due to resource constraints, we did not perform peer review for the search strategies.
[vii] The numbers of records identified from each database are indicated in the PRISMA flow chart (Figure 2).
